# Tumor-like appearance of Spermatic Granuloma

**DOI:** 10.1590/S1677-5538.IBJU.2018.0676

**Published:** 2019-07-27

**Authors:** Pablo Garrido-Abad, Ariel Díaz-Menéndez, Luis García-Martín, Isabel Senra-Bravo, Manuel Fernández-Arjona

**Affiliations:** 1 Department of Urology, Hospital Universitario del Henares, Coslada, Universidad Francisco de Vitoria, Madrid, Spain; 2 Department of Pathology, Hospital Universitario del Henares, Coslada, Madrid, Spain

## INTRODUCTION

The epididymis is an organ intimately attached to the posterolateral aspect of the testis. It is a complex tubular network that connects the testicular efferent ducts to the vas deferens and a variety of no neoplastic and neoplastic lesions involves the epididymis (1). Spermatic granuloma (SG) is a granulomatous lesion that presents clinically as a nodular lesion in the region of epididymis. It represents a chronic immune response to extravasated sperm caused by trauma, surgery or infection. There are only few documented cases of spermatic granuloma in the literature. We present a new case of SG.

## CASE REPORT

**Figure 1:**
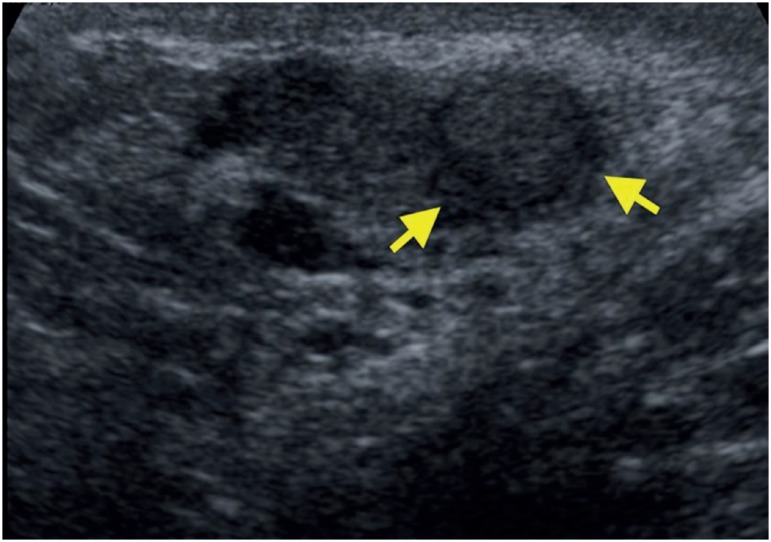
A 1.5 cm well-defined hypoechoic solid nodule at the right epididymis coil (yellow arrow).

**Figure 2:**
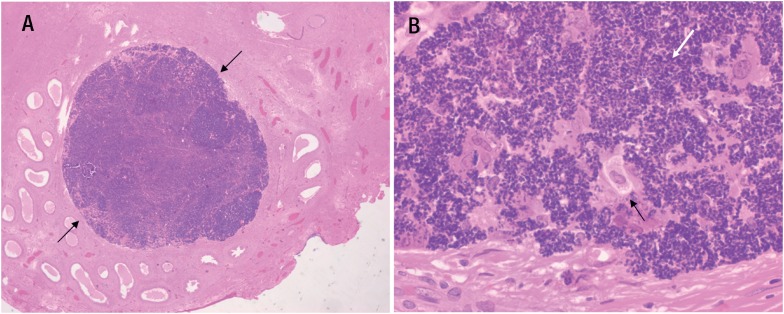
(A) H-E stain 10x sperm granuloma and H-E stain 20x, (B) phagocytosed sperms in the macrophages (black arrows) delimited by histiocytic reaction and giant cells (white arrow).

A 45-year-old man presented in the office with chronic pain in right testicle. He had history of closed ended vasectomy 7 years ago. Physical examination revealed a nodular firm lesion in the epididymal coil and ultrasound showed a 1.5 cm well-defined hypoechoic solid nodule at the right epididymis coil (Figure-1, yellow arrows). The laboratory data, including alpha fetoprotein, beta human chorionic gonadotropin and lactate dehydrogenase were normal. Suspected diagnosis was adenomatoid tumor, thus a testis-sparing surgical excision was performed and epididymal nodule was resected. A white nodule 1.7 cm in diameter was found in the resected specimen. Pathological findings were phagocytosed sperms in the macrophages delimited by histiocytes and giant cells (Figure-2), and scarce calcifications, with final diagnosis of SG.

## DISCUSSION

Various inflammatory, benign, and malignant lesions of epididymis have been described in the literature. Although only 3% of all solid extra testicular masses are malignant (2), previous studies have shown the malignancy rate for solid epididymal masses, to be as high as 16% (3). A SG is a rare benign condition, identified in only 5-7.5% (4, 5) of epididymal nodules. It was firstly described by Friedman (6) in 1949, tends to occur secondary to inflammation, trauma and vasectomy, and is thought to be a granulomatous reaction to extravasated sperm cells, characterized by initial influx of neutrophils that are replaced by histiocytes and multinucleated giant cells, which engulf the extravasated spermatozoa (7). The pathogenesis of SG is based on the tubular obstruction which results in an increased luminal pressure and leakage of sperm, where due to the highly antigenic nature of spermatozoa are founded extraluminally from the male ductal system and they are recognized as a completely foreign tissue. Its incidence after vasectomy ranges between 5-97%, located more frequently in the vas deferens than in the epididymis, being more common after open ended vasectomy and least often after electrocoagulation (8). Differential diagnosis includes tuberculous granulomatous inflammation, spermatocele, epididymo-orchitis, malakoplakia, adenomatoid tumor and primary testicular tumor (4). Scrotal sonography plays an important role in distinguishing solid masses from inflammatory masses of the epididymis. Recent studies have suggested an increased interest in the role of colour doppler, contrast-enhanced ultrasound, and magnetic resonance in diagnosis and management of scrotal lesions (9).

Small size, the presence of a hyperechoic or hypoechoic rim circumscribing the lesion and little or absent blood flow are important findings when comparing benign neoplastic lesions with inflammatory lesions (10). Local excision is still the gold standard for the treatment of epididymal nodules. However, in recent years, fine needle aspiration cytology has an important role in the differential diagnosis of epididymal nodules because they are easily accessible, providing adequate material for examination. This in the future could avoid unnecessary surgeries (4).
